# Extramedullary Plasmacytomas of the Nasal Cavity: Case-Based Perspectives into Optimizing the Diagnostic Differentiation from Inflammatory Polyps

**DOI:** 10.3390/medicina61081406

**Published:** 2025-08-01

**Authors:** Carmen Aurelia Mogoantă, Codruț Sarafoleanu, Andrei Osman, Irina Enache, Shirley Tarabichi, Constantin-Ioan Busuioc, Ilona Mihaela Liliac, Dan Iovanescu, Ionuţ Tănase

**Affiliations:** 1 Department of Otorhinolaryngology, University of Medicine and Pharmacy of Craiova, 200349 Craiova, Romania; carmen.mogoanta@umfcv.ro; 2 Department of Otorhinolaryngology, Faculty of Medicine, “Carol Davila” University of Medicine and Pharmacy, 050474 Bucharest, Romania; ionut.tanase@umfcd.ro; 3 Department of Otorhinolaryngology, “Sf. Maria” Clinical Hospital, 011172 Bucharest, Romania; 4 Department of Anatomy and Embriology, University of Medicine and Pharmacy of Craiova, 200349 Craiova, Romania; irina.enache@umfcv.ro; 5 Doctoral School, Faculty of Medicine, “Carol Davila” University of Medicine and Pharmacy, 050474 Bucharest, Romania; shirley.tarabichi@gmail.com; 6 Department of Pathology, Sfânta Maria Hospital, 011172 Bucharest, Romania; busuioc.constantin@gmail.com; 7 Department of Histology, University of Medicine and Pharmacy of Craiova, 200349 Craiova, Romania; ilona.liliac@umfcv.ro; 8 Department of Otorhinolaryngology, University of Medicine and Pharmacy Timișoara, 300041 Timisoara, Romania; dan.iovanescu@umft.ro

**Keywords:** sinonasal extramedullary plasmacytoma, unilateral nasal mass, differential diagnosis, solitary plasmacytoma, sinonasal tumors, head and neck oncology, histopathological diagnosis, radiotherapy outcomes, endoscopic tumor resection

## Abstract

*Background and Objectives*: Extramedullary plasmacytoma (EMP) is a rare monoclonal B-cell neoplasm that typically affects the head and neck region, with a predilection for the sinonasal tract. Clinical presentation is often nonspecific, leading to delayed diagnosis. This study aims to improve our understanding of sinonasal EMP by reviewing the recent literature and presenting a case series from our clinical experience. *Materials and Methods*: A systematic review of published cases of sinonasal EMP from 2000 to 2023 was conducted using the PubMed database, yielding 28 eligible cases. Additionally, we retrospectively analyzed three patients diagnosed and treated at our institutions. Inclusion criteria included histologically and immunohistochemically confirmed EMP without evidence of systemic multiple myeloma. Data on demographics, tumor location, symptoms, treatment, and outcomes were collected and analyzed descriptively. *Results*: Sinonasal EMP most commonly presented with unilateral nasal obstruction and epistaxis. Tumors were primarily located in the nasal cavity and paranasal sinuses, often extending beyond a single anatomical site. In the literature cohort, the most frequent treatment was combined surgery and radiotherapy (35.71%), followed by radiotherapy alone (17.86%). Recurrence was reported in 10.71% of cases, and 7.14% of patients died due to disease progression. All three patients in our case series underwent surgical excision; two received postoperative radiotherapy. No recurrences or progression to multiple myeloma were observed during follow-up (12–24 months). *Conclusions*: Sinonasal EMP is a rare but radiosensitive tumor with a favorable prognosis when treated with surgery and/or radiotherapy. Early diagnosis, histopathological confirmation, and exclusion of systemic disease are essential. Multidisciplinary management and long-term follow-up are critical due to the risk of recurrence and transformation into multiple myeloma.

## 1. Introduction

Plasmacytoma is a type of neoplasm that arises from monoclonal B cells. There are various types of plasmacytoma: solitary plasmacytoma of bone (SPB), extramedullary plasmacytoma (EMP), multiple myeloma (MM), and lymphoplasmacytic lymphoma.

EMP was initially described by Schridde and colleagues in the year 1905 [1].

EMP is a rare condition that is defined as a localized plasma cell lesion that develops in tissues outside of bone marrow plasmacytosis [2].

EMP is a very rare tumor, representing 3–5% of all plasma neoplasms [3].

EMP can occur in any part of the body; however, the head and neck region is the predominant location, with approximately 80–90% of cases occurring at this level. The nasal cavity and paranasal sinuses are most commonly involved, followed by the palatine tonsils and oral cavity. The symptomatology is not specific and may include headache, epistaxis, nasal obstruction, nasal discharge, dysphagia, and sore throat [4,5,6].

EMP may result from inhalation of chemicals, viral infection, excessive irradiation, and genetic abnormalities that affect the reticuloendothelial system [7].

The clinical presentation of EMP is nonspecific and often overlaps with benign or inflammatory sinus conditions. Radiological imaging (CT and MRI) is indispensable for the evaluation of the lesion and the examination of bone involvement and local invasion, as well as for the planning of the surgical approach. CT is particularly useful for finding changes in bone structure or destruction. MRI, on the other hand, gives information about soft tissue, which helps define tumor edges and clarify the difference between normal tissue and inflammatory changes that are nearby. Also, radiological imaging is essential for the subsequent surveillance of recurrence or progression to multiple myeloma [5].

EMP is typically diagnosed based on histological and immunochemical exams that highlight markers such as Vs38c, CD138, CD20, CD38, CD 56, CD79a, CD117, lambda light chains (LLCs), and kappa light chains (KLCs). CD138 can be identified as the marker that is used most frequently to identify plasma cells [8,9].

An EMP may be classified as primary when it arises de novo or as secondary when it develops during the progression of multiple myeloma (MM). Extramedullary disease has the potential to progress to MM in 17–33% of cases [10,11,12]. The transformation of EMP into MM can occur in 8–31% of cases, as described in the literature [8,13,14].

For the management of EMP, there are no clear guidelines, and the majority of the experience has been collected from a number of different case reports that have been published over the course of several decades. However, in the case of sinonasal EMP, radiotherapy is frequently the preferred option because of the increased sensitivity of EMP to radiation. Surgical excision in association with radiotherapy has demonstrated the most favorable survival rates. The efficacy of chemotherapy in the treatment of sinonasal EMP remains unclear [15].

This study includes an overview of the documented cases of extramedullary plasmacytoma of the sinonasal region found in the literature published between 2000 and 2023 and a separate analysis describing our experience, presenting 3 three clinical cases.

## 2. Materials and Methods

**Figure 1:**
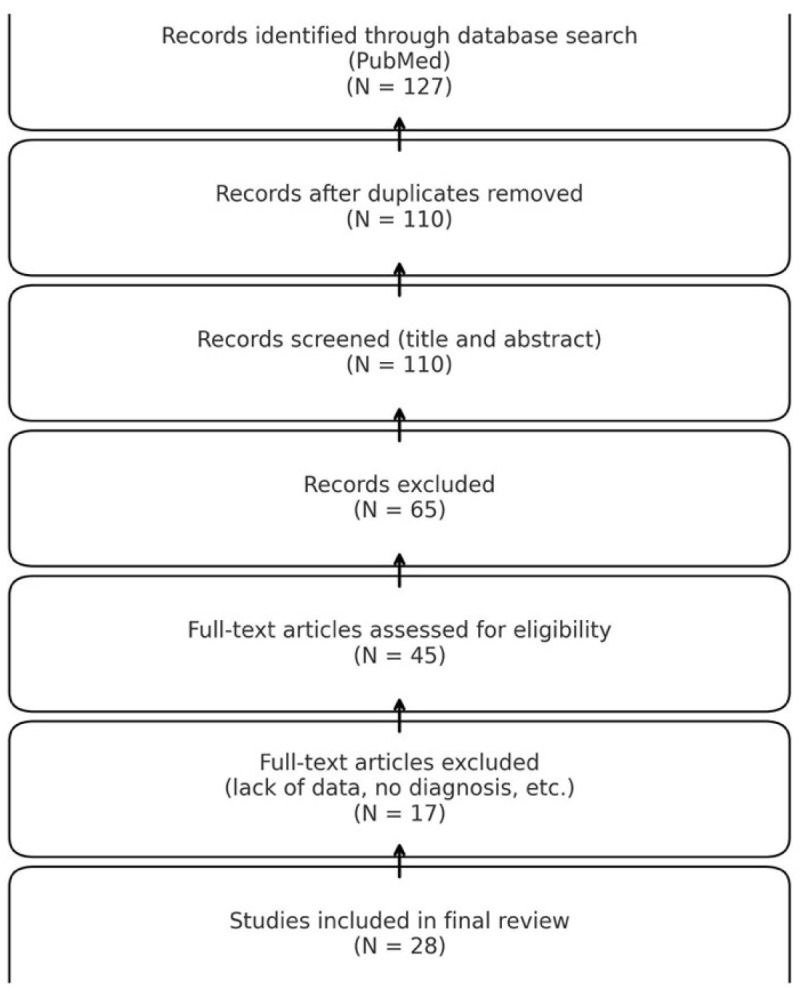
The selection process represented in a PRISMA diagram.

We performed an extensive literature search by accessing the PubMed database, which identified all English- and non-English-language manuscripts on sinonasal EMP published between January 2000 and December 2023. Our search criteria included keywords such as “sinonasal plasmacytomas,” “sinus plasmacytomas,” and “nasal plasmacytomas,” which initially yielded 127 articles. Duplicates were removed, and additional filters were applied to exclude non-English-language publications and non-human studies, resulting in a refined selection of 110 articles. We subsequently screened titles and abstracts to exclude studies that were not case-based or lacked clinical relevance, reducing the dataset to 45 full-text articles. After full-text evaluation, 17 studies were excluded due to insufficient individual clinical data or unclear histopathological confirmation. A total of 28 studies met the inclusion criteria and were included in the final analysis. The inclusion and exclusion criteria applied are summarized in Table 1. The selection process is summarized in the PRISMA flow diagram (Figure 1).

This paper gives a complete overview of the sinonasal EMP cases reported in the last 23 years.

We included only human studies written in the English language that provided individual data on sinonasal plasmacytomas, encompassing details on diagnosis, treatment, follow-up, and outcomes.

We extracted outcome measures that covered various aspects, such as demographic information (gender, age), tumor location, presenting symptoms, radiographic imaging, the primary mode of treatment, adjuvant therapy, instances of recurrence, metastasis, development of multiple myeloma, follow-up duration, secondary treatment for recurrence or metastasis, and overall survival. We summarize all the extracted data in Table 2.

We used descriptive statistics to analyze the available data for statistical purposes. Descriptive analysis of characteristics including gender, tumor location, symptomatology, treatment modalities, and outcomes highlighted frequency and percentage distributions. Categorical variables were summarized with absolute and relative frequencies, while continuous variables, such as age, were characterized by means and standard deviations. All analyses were performed using Microsoft Excel.

Following surgical treatment, the sample was rinsed with phosphate-buffered saline (PBS), and tissue fragments were fixed in 10% neutral buffered formalin for 24 h at room temperature. The specimens were then processed for paraffin embedding. Tissue sections of 3–4 μm thickness were mounted on glass slides and stained with Hematoxylin–Eosin (HE) to evaluate histological characteristics. Serial sections of 3–4 μm thickness were dewaxed and rehydrated. Antigen retrieval was performed by incubating the sections in a microwave oven using an appropriate buffer. To block endogenous peroxidase activity, the sections were treated with 3% hydrogen peroxide (H_2_O_2_) in methanol.

After a blocking step to minimize nonspecific binding, the sections were incubated overnight at 4 °C with one of the primary antibodies listed in Table 1. The next day, the sections were washed with phosphate-buffered saline (PBS) at pH 7.4–7.6 and subjected to immune signal amplification using the Dako Envision™+ Dual Link System–Horseradish Peroxidase (HRP) (Dako, Carpinteria, CA, USA), according to the manufacturer’s instructions. Finally, the slides were counterstained with Mayer’s Hematoxylin.

For each antibody, a negative control was included by substituting the primary antibody with 10 mM PBS at pH 7.4–7.6. Color development was carried out using 3,3′-Diaminobenzidine (DAB) tetrahydrochloride (Sigma-Aldrich, St. Louis, MO, USA) in the presence of hydrogen peroxide (H_2_O_2_) (Merck). Nuclear counterstaining was performed with Mayer’s Hematoxylin. Finally, the sections were mounted using DPX mounting medium (Sigma-Aldrich).

For the IHC study, we used the following primary antibodies for both positive and differential diagnosis: (anti-human CD20cy, clone L26, Dako, dilution 1:100; anti-human, CD79-α, clone JCB117, Dakoz, dilution 1:50; anti-S100,rabbit polyclonal, Dako, dilution 1:50, antigen retrieval:citrate buffer, pH 6; anti-EMA, mouse monoclonal, Dako, dilution 1:50, antigen retrieval:citrate buffer, pH 6; anti-synaptophysin, mouse monoclonal, Dako, dilution 1:20, antigen retrieval: citrate buffer, pH 6; anti-CK AE1/AE3, mouse monoclonal, Dako, dilution 1:50, antigen retrieval: citrate buffer, pH 6; anti-CD45/LCA/CLA, mouse monoclonal, Dako, dilution 1:50, antigen retrieval: citrate buffer, pH 6; anti-human; CD138 (Syndecan-1), rabbit monoclonal, clone A23248, ABclonal, dilution 1:100, antigen retrieval: citrate buffer, pH 6).

## 3. Results

### 3.1. Literature Data Analysis

There were 28 cases of SN-EMP included in this study (Table 1). The average age at diagnosis was 45.07 years old (SD  ±  19.70). The majority of patients were aged between 40 and 70 years, with 15 (53.57%) males and 13 (46.42%) females (Table 3).

Among the cohort of 28 patients, tumor localization predominantly affected the nasal cavity and paranasal sinuses, occurring in 22 (78.57%) of the patients. Only 3 (13.63%) nasal cavity tumors were localized to that area, while 19 (86.37%) exhibited extension beyond it. In the same way, paranasal sinuses were implicated in 22 (78.57%) of the cases, with only 5 (22.72%) cases limited to the paranasal sinuses—3 cases confined to the maxillary sinus and 2 to the sphenoid sinus. Nasopharyngeal involvement was noted in seven (25%) patients; however, only two (28.57%) were limited to the nasopharynx, while five (71.42%) exhibited extension beyond this region (Table 4).

The most common treatment in the reviewed literature was surgery and radiotherapy, used in 35.71% of the cases, followed by radiotherapy alone, in 17.86%. Surgery alone represented 14.29% of the cases; radiotherapy with chemotherapy and a combination of radiotherapy, surgery, and chemotherapy were used 10.71% of the time. Chemotherapy alone was rare, used in 3.57% of the cases, and 7.14% received no treatment (Table 5).

The most frequent symptomatology was unilateral epistaxis, followed by nasal obstruction.

At follow-up, 67.89% were disease-free and showed no tumor recurrence, 10.71% relapsed, and 7.14% died. Follow-up data was not available for two patients (7.14%) diagnosed with multiple myeloma, as well as for two additional patients (7.14%) who declined treatment, as presented in (Table 6).

### 3.2. Presentation and Analysis of Our Cohort

The patients were diagnosed with EMP by meeting the following criteria: the presence of one or more extramedullary plasma cell tumors, normal plasma cell density in the bone marrow with minimal morphological alterations or plasma cell infiltration below 10%, absence of radiological evidence of osteolysis, absence of hypercalcemia or renal failure, and low or absent serum M-protein levels.

#### Case Report

CASE 1

**Figure 2:**
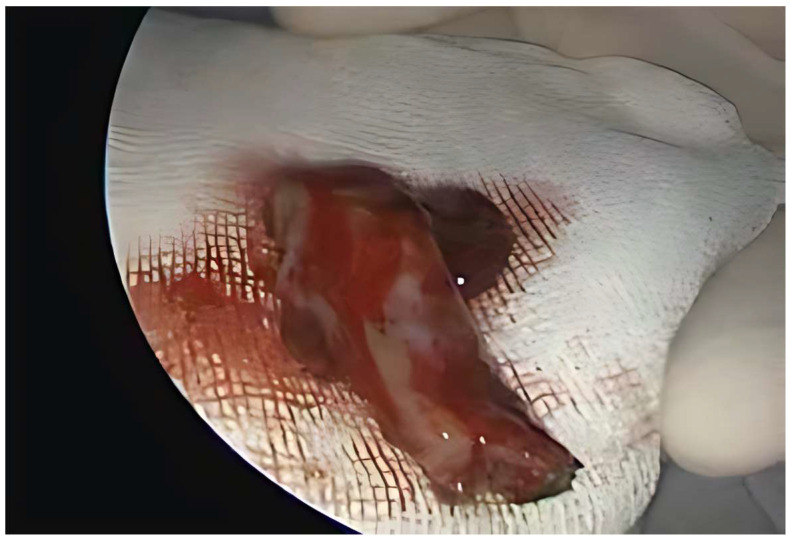
The resected specimen.

A 43-year-old male patient presented with bilateral nasal obstruction, posterior rhinorrhea, and recurrent epistaxis, symptoms that had begun 3 months ago and had progressively worsened. The ENT clinical examination and nasal endoscopy revealed an infiltrating, vegetative mass on the left nasal fossa and nasopharynx, measuring approximately 2 cm, reddish in color, mildly tender, and bleeding easily, without any pathological secretions. No lymphadenopathy was found, and his blood test results, including hemoglobin levels, were within normal limits. Images obtained from an MRI scan of the head revealed a mass of soft tissue in the nasopharynx and left nasal fossa. The mass did not cause any bone destruction. It was decided that the mass should be surgically removed, so, under general anesthesia and endoscopic control, the tumor was extirpated “en bloc” (Figure 2).

**Figure 3:**
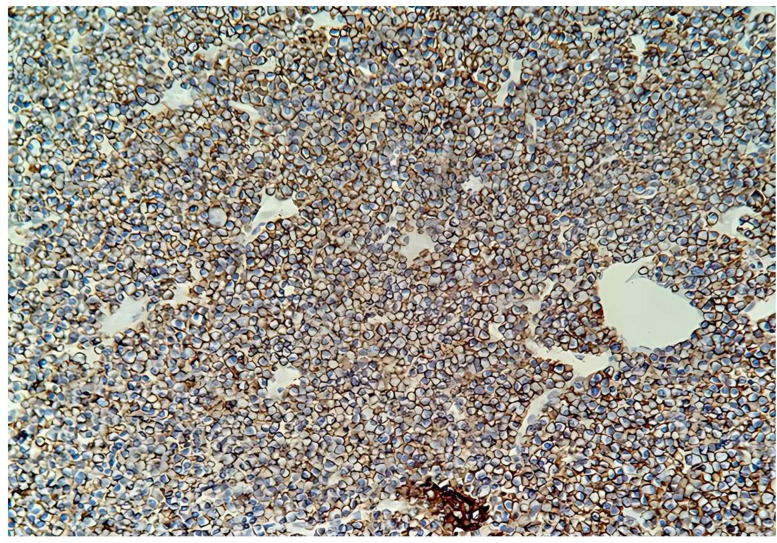
CD 138 is positive in the plasma cells on the cell membrane. (IHC 20×).

The resected tumor specimen was sent for histopathological examination. Pathological examination revealed diffuse tumor infiltration resembling plasmacytoid cells, accompanied by hemorrhagic areas and focal ulceration of the overlying epithelium. On immunochemistry examination, the tumor cells expressed strong immunoreactivity with CD38 and CD138 (Figure 3).

This established the diagnosis of EMP of the nasopharynx. The patient declined all forms of oncological therapy, including radiotherapy treatment. One year postoperatively, the patient underwent control imaging (MRI of the head and neck) and an ENT exam, which did not reveal signs of recurrence.

CASE 2

**Figure 4:**
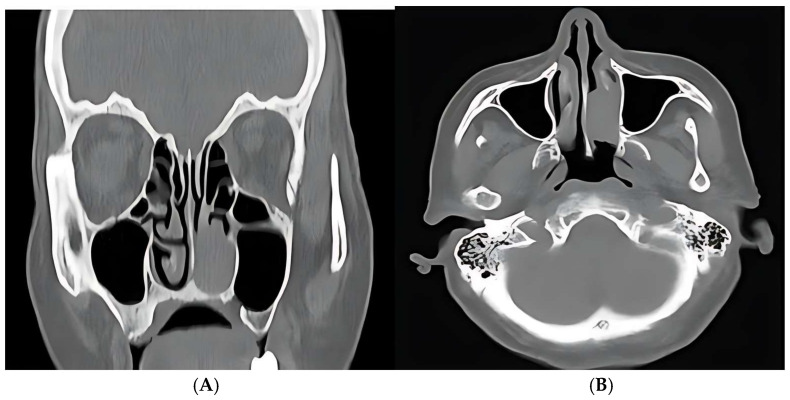
Paranasal CT scan images, coronal (**A**) and axial (**B**) view, showing a soft tissue mass in the left nasal cavity, with no bone invasion or local extension.

A 79-year-old female patient presented with progressive left nasal obstruction and repeated episodes of left unilateral epistaxis at minimal effort. The symptomatology of the patient started 2 years before the presentation. The patient did not have major comorbidities, and routine blood examinations were within normal limits. ENT clinical examination and nasal endoscopy revealed a sessile, reddish mass, slightly bleeding upon instrumental palpation, located in the left nasal fossa, which almost completely obstructed it. The CT scan of the paranasal sinuses revealed a soft tissue mass in the left nasal cavity, arising from the left inferior turbinate, without bone invasion or local extension (Figure 4).

**Figure 5:**
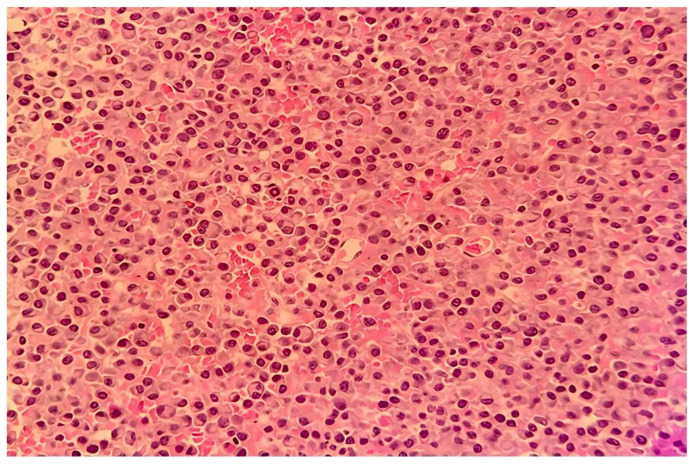
Atypical plasma cells, some with bi-nucleation and atypical mitoses (HE 20×).

It was decided that the mass be removed surgically. Consequently, the tumor was extirpated “en bloc” under endoscopic control and general anesthesia and then sent for histopathological examination. Histopathological examination showed diffused plasmacytoid tumor cells in the stroma along with areas of necrosis and hemorrhage and cells with bi-nucleation and atypical mitoses (Figure 5).

Immunohistochemical examination evidenced positive expressions of CD79a, CD138, and CD56. This led to the diagnosis of EMP in the left nasal cavity. After the diagnosis was established, for better disease control, the patient underwent local radiotherapy (40 Gy over a period of 3 weeks). On follow-up after 2 years of completed oncological care, nasal endoscopy and CT scan examination of the head showed no signs of recurrence.

CASE 3

**Figure 6:**
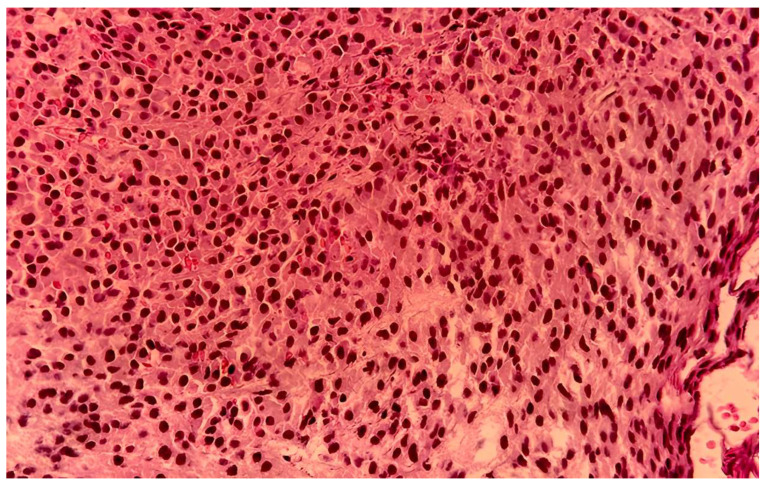
Compact mass of cells with abundant eosinophilic cytoplasm and eccentrically located nuclei and some cells with a spindled morphology (HE 20×).

A 47-year-old male patient presented with unilateral (right) nasal obstruction, which had been present for over one year. The patient had no comorbidities, and blood tests were within normal limits. ENT examination and nasal endoscopy revealed a mass in the right nasal cavity that completely obstructed the ipsilateral osteomeatal complex and purulent secretions. The mass bled on instrumental palpation. CT examination of the paranasal sinuses revealed a soft tissue mass that obstructed the entire right nasal fossa, extended into the right ethmoid sinus, and opacified the right maxillary sinus. The patient underwent endoscopically guided surgery under general anesthesia for tumor removal. The specimen was sent for histopathological examination, which showed a compact mass of cells with abundant eosinophilic cytoplasm and eccentrically located nuclei and some cells with a spindled morphology (Figure 6).

The immunochemistry examination showed that the tumor cells were positive for CD138. The patient also underwent oncological treatment—radiotherapy with 44 Gy over a period of 1 month. The endoscopic exam and CT scan of the paranasal sinuses showed no signs of relapse at the 18-month follow-up.

In all of our three patients presented in the study, we also performed an extensive evaluation (serum protein electrophoresis and bone marrow examination) to establish whether they also had associated multiple myeloma. These investigations were negative in all the patients.

Table 7 presents a systematic summary of our institutional case series, encompassing clinical presentation, pathological characteristics, treatment modalities, and follow-up information for each patient.

## 4. Discussion

The published literature on EMP of the nasal cavity and paranasal sinuses is limited to case reports or small series due to the rarity of the condition. The present study includes our experience with three patients and a literature review of twenty-eight cases.

EMP originates predominantly in the head and neck region, primarily affecting the elderly, with a peak incidence in the 6th and 7th decades of life. A total of 44% of cases of EMP of the head and neck are located in the nasal cavity/paranasal sinuses, while 18% involve the oropharynx [2,19,20,21,22]. EMP appears to have a male predilection (male/female ratio = 3:1) and it was first described by Schridde in 1905 [1]. Among the patients included in our study, EMP was more frequently observed in males, with a male-to-female ratio of 2:1 and a median age of 56.3 years [23,24,25,26,27]. Most of affected patients generally receive endoscopic nasal surgery as primary means of treatment [27,28,29,30]. These rare cases bring forth diagnostic challenges posed by EMP, as they often mimic other neoplastic lesions [31,32,33]. Treatment across the cases commonly involves radiotherapy for main-lesion control [34]. Surgical resection usually provides an adequate biopsy material and restores nasal functionality but it also serves a strong emphasis on monitoring for potential progression to multiple myeloma or local disease recurrence after radiotherapy [30,31,32].

In the head and neck region, about 80–90% of EMP cases are located in the sinonasal cavity [35,36].

In the cohort of 28 patients with sinonasal EMP analyzed, tumor localization showed an individual pattern of frequent involvement of the nasal cavity and paranasal sinuses, with a tendency for extension beyond a singular anatomical site in most cases. In 22 patients, we observed that tumors involving the nasal cavity were the most frequent. The majority of cases (19 out of 22) showed extension of the tumor beyond the nasal cavity, and only 3 cases indicate a localized disease process limited to the nasal cavity.

Paranasal sinus involvement was similarly common, observed in 22 of the 28 patients. In this group, three lesions were restricted exclusively to the maxillary sinus, while two were limited to the sphenoid sinus. The remaining cases indicated either multi-sinus involvement or extension beyond the paranasal sinuses into the nasal cavity or adjacent regions.

In seven cases, the nasopharynx was determined to be the primary location of involvement. Additionally, in three cases, the tumor was limited only to the nasopharyngeal region, while the other four cases presented evidence of dissemination beyond the nasopharynx.

Symptomatology is insidious, and this might lead to a delayed presentation, resulting in a late diagnosis by the physician and advanced stages of the disease. The symptoms refer to the location of the tumor more than its characteristics. Some of the presentation symptoms include localized edema, unilateral nasal obstruction, recurrent unilateral epistaxis, rhinorrhea or adenopathy, facial pain, proptosis, perforated nasal septum, and nasal pyramid deformity.

The most frequent manifestations of the disease, as reported in both the literature and our cases, are nasal obstruction and recurrent epistaxis [36].

In a group of 20 patients with sinonasal EMP, Kapadia and colleagues [37] noticed that the following symptoms were the most common: tumor or local edema in 80%, nasal obstruction in 35%, epistaxis in 35%, localized pain in 20%, proptosis in 15%, rhinorrhea in 10%, regional lymphadenopathy in 10%, and paralysis of the VI cranial nerve in 5% of cases.

In our analyses, the results are similar to the data reported by others. All of our three patients presented with unilateral nasal obstruction, and two of them also experienced epistaxis. In the 28 cases from the literature that we studied, unilateral epistaxis was the most common symptomatology, followed by nasal obstruction.

Paraclinical exams such as nasal endoscopy and radiological investigations are nonspecific; they are used to assess the lesion. The MRI examination may reveal a mildly heterogeneous signal that appears hyperintense on T2 and intermediate on T1, while various levels of intensity can also be observed on the craniofacial CT examination after the administration of contrast substance. Current international guidelines indicate that CT and MRI are appropriate for initial staging, and whole-body PET-CT is helpful for evaluating the disease at any other site, and it also helps in monitoring the patient’s response to treatment [38].

Histopathological examination reveals a dense, homogeneous proliferation of plasma cells arranged in cords, solid masses, or chains, accompanied by nuclear atypia characterized by round or oval nuclei, vesicular nuclear chromatin, and mitotic activity [9,39,40]. Immunochemical analysis indicates a uniform infiltration of monoclonal plasma cells, typically expressing CD138 and/or CD38 on their surface [41]. The immunophenotypic profile in our series—particularly the expression of CD138, CD38, and, in one case, CD56—is concordant with characteristic markers of plasmacytic differentiation described in the literature [8]. The presence of CD56, although variable across studies, has been implicated as a potential prognostic marker; its expression in one of our cases underscores the heterogeneity of EMP and the need for broader immunohistochemical assessment in clinical practice [8,9].

It is important to note that certain immunohistochemical markers may provide prognostic insights in EMP. Among these, CD56 has garnered particular interest. Studies have shown that the absence of CD56 expression may be associated with more aggressive clinical behavior and an increased risk of progression to multiple myeloma, particularly in cases with extramedullary involvement [8,9]. Furthermore, a meta-analysis has supported the prognostic relevance of CD56, indicating that its expression status could guide risk stratification and long-term surveillance strategies [8]. In our institutional series, one case demonstrated CD56 positivity, emphasizing the need for standardized immunohistochemical evaluation in all patients with EMP.

Up to 20–30% of cases of EMP have the potential to progress to MM, and it is still impossible to determine which cases of EMP might do so based on paraclinical investigations.

To diagnose EMP and differentiate it from MM, histopathological and immunohistochemical examinations must be performed, along with the fulfillment of specific criteria [42,43]: (1) the presence of one or more extramedullary plasma cell tumors; (2) bone marrow smear with a normal ratio of plasma cells in the bone marrow, including no observed plasma cell morphology or a plasma cell ratio of less than 10%; (3) absence of radiological evidence indicating osteolysis; (4) absence of hypercalcemia or renal insufficiency; (5) absence or minimal M-protein serum concentration.

After confirmation of EMP, additional investigations must be conducted, including bone marrow evaluation, serum protein electrophoresis, complete blood count, renal function evaluation (eGFR), and skeletal examination to exclude MM [17].

In our cases, bone marrow evaluation and laboratory serological tests evidenced the absence of bone marrow involvement, no hypercalcemia or renal failure, and the immunoglobulin electrophoresis within normal parameters, which confirmed the absence of MM. From the 28 cases, 2 (7.14%) developed into MM.

The differential diagnosis of nasal EMP includes pathologies like lymphoma, melanoma, inverted papilloma, and sinonasal fungal material, and also rhinoscleroma, olfactory neuroblastoma, and pituitary adenoma [30,44]. From a histopathological perspective, the distinction between EMP and multiple myeloma is the most difficult. However, the absence of bone lysis, normal protein electrophoresis, and the absence of anemia are the distinguishing features between the two tumor types [45,46].

The treatment of nasal EMP requires the development of a treatment plan in association with the oncology team. This plan should be personalized based on the histological type of the tumor, its stage, the feasibility of complete resection, the patient’s medical condition, the risks of the treatment, and comorbidities. The surgical team’s expertise in managing potential intraoperative complications, the patient’s preferences, and the available reconstructive options should also be taken into account.

In this context, endoscopic nasal surgery serves as a valuable tool, offering a minimally invasive and safe approach that allows for both diagnostic procedures—such as obtaining tissue for histopathological and immunohistochemical evaluation—and therapeutic intervention through complete tumor excision with clear margins when anatomically feasible [47,48].

EMP is a radiosensitive tumor, so the first recommended therapeutic method is radiotherapy when the surgery cannot be performed. Radiotherapy offers a local control rate of 90–100%. Regarding the optimal recommended dose, there is no consensus [49,50].

Advancements in radiotherapy have significantly improved treatment precision and outcomes in EMP, especially for tumors in anatomically complex regions such as the upper aerodigestive tract. Intensity-modulated radiation therapy (IMRT) and stereotactic body radiotherapy (SBRT) allow for conformal dose delivery while sparing adjacent critical structures. While no universal consensus exists regarding the optimal dose, the current literature supports total radiation doses ranging from 40 to 50 Gy, with dose escalation up to 60–70 Gy in selected cases of bulky or incompletely resected disease (Holler et al., Cancer Med. 2022) [15,19,47].

Polymodal treatment, which includes extensive surgery followed by radiotherapy, is also described in the literature and is considered to offer the most effective therapeutic results [39,47,51].

Alexiou et al. [34] found that in the treatment of head and neck EMP, the combination of radiation and surgery resulted in an increase in 5-year survival of approximately 50% and they recommended surgery followed by RT for EMP when complete resection is difficult to achieve.

In a study of 68 patients with head and neck EMP, Bachar et al. [13] analyzed the rates of local recurrence, regional recurrence, and progression to MM after RT alone and after simple surgery. They found that surgery without RT decreased the recurrence rate at 5 years of follow-up from 82% to 75%. These authors recommend that RT be considered primary therapy and that postoperative RT be applied to patients with involved surgical margins but is not necessary for those who underwent complete surgical excision with negative margins.

A potential explanation for these statistical findings lies in the fact that the therapeutic efficacy of radiotherapy alone, as opposed to its combination with chemotherapy, is influenced by several tumor-specific factors, including histological subtype, tumor size, and anatomical location. Chemotherapy is not typically regarded as a first-line therapeutic option for EMP, given the high radiosensitivity of these tumors. Radiotherapy alone constitutes the standard of care in the majority of cases, particularly when complete surgical resection is impractical. The use of chemotherapy is generally reserved for select clinical contexts, such as cases with extensive tumor burden, resistance to local therapy, refractory disease, or progression to multiple myeloma [15,47].

Overall, the studies suggest that combined-modality treatment—surgery followed by adjuvant radiotherapy—offers the most effective local control for EMP [15,17].

According to prior studies, EMPs measuring less than 5 cm demonstrate excellent local control when treated with a radiation dose of 40 Gy administered over 20 fractions. In contrast, it is recommended that lesions exceeding 5 cm in size, which are associated with an increased risk of recurrence, receive a higher dose in the range of 50 Gy delivered over 25 fractions [8,41]

However, a definitive evaluation of treatment efficacy can only be made several weeks following the completion of radiotherapy, as plasmacytomas characteristically exhibit a delayed response to radiation.

Long-term surveillance by a multidisciplinary team including otolaryngologists, radiologists, and oncologists in patients diagnosed with EMP is essential due to the risks of local recurrence and potential transformation into MM. Current guidelines recommend that during the first two years, patients should be evaluated every three to six months. From three to five years, evaluations should be performed every six to twelve months. After the first five years, patients are evaluated annually for life [52].

The follow-up protocol includes a clinical ENT examination with nasal endoscopy, hematological evaluations (including complete blood count, serum calcium, creatinine, and serum protein electrophoresis), and imaging evaluation (CT or MRI). In certain cases, or if relapse or development of multiple myeloma is suspected, PET/CT and a subsequent bone marrow evaluation should be performed.

Certain prognostic factors, such as larger tumor size (>5 cm), persistence of M-protein, negative CD56 expression, and incomplete response to initial therapy, have been associated with an increased risk of progression and should prompt more intensive monitoring [53,54].

In our cases, all patients underwent tumor resection by surgery, and two of them also received radiotherapy. All of three patients did not show any relapse at the follow-up (12–24 months). Consistent with previously published data, all patients in our series demonstrated favorable local control following either surgical resection alone or in combination with radiotherapy, aligning with reported local control rates exceeding 90% for appropriately treated EMP. In some larger series, tumor recurrence or progression to multiple myeloma is observed in up to 30% of cases over time [55]. During the follow-up period, none of our patients exhibited local recurrence or progression to multiple myeloma [56].

In the reviewed literature, 10 patients (35.71%) underwent combined surgery and radiotherapy. Radiotherapy was administered exclusively in five cases, constituting 17.86% of the total. Four patients (14.29%) underwent surgery exclusively, another three cases (10.71%) received radiotherapy combined with chemotherapy, and three patients (10.71%) received a combination of surgery, radiotherapy, and chemotherapy. There was one patient who received chemotherapy on its own (3.57%), and two patients (7.14%) did not receive any treatment at all. At follow-up, 67.86% of patients were alive and disease-free. Recurrence was observed in 10.71% of the patients, while 7.14% died due to the disease. It is not mentioned in the articles whether follow-up was performed on the two patients (7.14%) who were also diagnosed with multiple myeloma and on the two (7.14) that refused any type of treatment.

Two patients with recurrence received only radiotherapy, while another underwent a combination of radiotherapy and chemotherapy. One patient who died of the disease underwent surgery and chemotherapy, while the other received a combination of radiotherapy, surgery, and chemotherapy.

Among the analyzed cases, patients who underwent both surgery and radiotherapy did not experience any recurrence of the tumor during the follow-up period. This highlights that this method is more effective than other alternative treatment protocols.

## 5. Conclusions

Sinonasal extramedullary plasmacytoma is a rare entity that presents with nonspecific clinical manifestations, such as unilateral nasal obstruction and epistaxis, leading to a delay in patient presentation and diagnosis.

Histopathological and immunohistochemical examinations are essential for diagnosis, and in conjunction with the results of other laboratory investigations—normal bone marrow smear, absence of osteolysis, no hypercalcemia or renal insufficiency, and absence or minimal M-protein serum concentration—evaluate systemic involvement to exclude multiple myeloma.

Due to the rarity of EMP, the nonspecific presentation, and nonspecific radiological findings, we consider that treatment strategies should be individualized. Based on our experience, which includes 3 patients and a review of 28 cases from the literature, we considered that radiotherapy in association with surgery when complete excision of the tumor with safe margins is possible is the preferred therapeutic modality, but radiotherapy alone may also be considered.

Follow-up is essential in cases of EMP due to the potential for relapse and development of multiple myeloma. Multidisciplinary management involving otolaryngologists, oncologists, radiation therapists, and pathologists is essential to improve outcomes in patients with sinonasal EMP.

## Figures and Tables

**Table 1:** Inclusion and exclusion criteria.

Inclusion Criteria	Exclusion Criteria
Original articles published in English (January 2000 and December 2023)	Articles not published in English
Histopathologically confirmed EMP	Non-human or animal studies
Human subjects only	Review articles, editorials, or conference abstracts without case-level data
Clinical details on diagnosis, treatment, follow-up, and outcomes	No histopathological confirmation of EMP
	Reports missing clinical information or follow-up information
	Studies that grouped sinonasal EMP cases with other head and neck locations

**Table 2:** Extracted data presentation.

Case No./Ref.	Gender/Age	Symptoms	Localization	Treatment	Outcome on Follow-Up	Multiple Myeloma
1/[1]	M/20	No symptoms	Nasal cavity	Surgery	No recurrence	No
2/[1]	M/48	Epistaxis	Nasal cavity	Surgery + RT	No recurrence	No
3/[1]	M/60	Epistaxis	Nasal cavity	RT	Recurrence	No
4/[2]	M/61	Epistaxis Facial pain Rhinorrhea	Nasal cavity Maxillary sinus	RT + CHT	Recurrence	No
5/[2]	M/60	Nasal obstruction Epistaxis Rhinorrhea	Nasal cavity Ethmoid sinus	RT	Recurrence	No
6/[2]	F/37	Nasal obstruction	Nasal cavity Maxillary sinus	CHT	No recurrence	No
7/[3]	M/75	Epistaxis	Nasopharynx	RT	No recurrence	No
8/[4]	F/44	Epistaxis	Nasal cavity Maxillary sinus	Surgery	No recurrence	No
9/[5]	M/32	Change/loss of vision	Sphenoid sinus	Surgery + RT	No recurrence	No
10/[6]	M/56	Nasal obstruction Epistaxis Facial pain and swelling	Nasal cavity Maxillary sinus Ethmoid sinus Sphenoid sinus nasopharynx	CHT	Died of the disease	No
11/[7]	F/67	Nasal obstruction Epistaxis Rhinorrhea	Nasal cavity Ethmoid sinus	Surgery + RT	No recurrence	No
12/[8]	F/75	Nasal obstruction	Nasal cavity Maxillary sinus	Surgery + RT	No recurrence	No
13/[9]	F/32	Nasal obstruction Epistaxis Facial pain and swelling Rhinorrhea Chance/loss of vision	Nasal cavity Maxillary sinus Ethmoid sinus Frontal sinus	Surgery + RT	No recurrence	No
14/[10]	F/87	Epistaxis	Nasal cavity Maxillary sinus, Sphenoid sinus Frontal sinus Nasopharynx	RT	No recurrence	No
15/[11]	F/50	Epistaxis Rhinorrhea Facial swelling	Nasal cavity Maxillary sinus Ethmoid sinus	Surgery + RT + CHT	-	Yes
16/[12]	M/24	Nasal obstruction Facial swelling and pain Rhinorrhea proptosis	Maxillary sinus	Surgery + RT	No recurrence	No
17/[13]	F/16	Nasal obstruction Epistaxis	Nasal cavity Maxillary sinus Ethmoid sinus Sphenoid sinus	Surgery + RT	No recurrence	No
18/[13]	F/26	Nasal obstruction Facial swelling	Maxillary sinus	Surgery	No recurrence	No
19/[13]	F/52	Nasal obstruction Epistaxis	Nasal cavity Nasopharynx	Surgery + RT	No recurrence	No
20/[13]	F/15	Epistaxis Facial swelling	Nasal cavity Maxillary sinus Sphenoid sinus	Surgery + RT + CHT	Died of the disease	No
21/[13]	M/23	Nasal obstruction epistaxis	Nasal cavity Maxillary sinus Ethmoid sinus	Surgery	No recurrence	No
22/[13]	F/15	Facial swelling	Maxillary sinus	No treatment	-	-
23/[14]	M/51	Nasal obstruction Change/loss of vision Headache	Nasal cavity Maxillary sinus	RT + CHT	-	Yes
24/[15]	M/31	Nasal obstruction Rhinorrhea Facial swelling	Nasal cavity Maxillary sinus, Ethmoid sinus, Sphenoid sinus Nasopharynx	RT	No recurrence	No
25/[15]	M/60	Nasal obstruction Epistaxis	Nasal cavity Nasopharynx	Surgery + RT	No recurrence	No
26/[16]	F/54	Change/loss of vision Headache	Sphenoid sinus	Surgery + RT	No recurrence	No
27/[17]	M/42	Nasal obstruction Headache	Nasal cavity Maxillary sinus	No treatment	-	-
28/[18]	M/49	Facial swelling and pain proptosis	Maxillary sinus Frontal sinus	Surgery + RT + CHT	No recurrence	No

**Table 3:** Demographic characteristics.

Parameter	Value
Total cases	28
Mean age at diagnosis (±SD)	45.07 years (±19.70)
Sex distribution	15 males (53.57%), 13 females (46.42%)

**Table 4:** Tumor localization.

Site	Cases (%)
Nasal cavity and paranasal sinuses	22 (78.57%)
-Localized only in nasal cavity	3 (13.63%)
-With extension beyond nasal cavity	19 (86.37%)
Paranasal sinus involvement	22 (78.57%)
-Localized only in sinuses	5–3 maxillary, 2 sphenoid (22.72%)
Nasopharyngeal involvement	7 (25%)
-Localized only in nasopharynx	2 (28.57%)
-With extension beyond nasopharynx	5 (71.42%)

**Table 5:** Treatment modalities.

Treatment	Cases (%)
Surgery + Radiotherapy	10 (35.71%)
Radiotherapy alone	5 (17.86%)
Surgery alone	4 (14.29%)
Radiotherapy + Chemotherapy	3 (10.71%)
Surgery + Radiotherapy + Chemotherapy	3 (10.71%)
Chemotherapy alone	1 (3.57%)
No treatment	2 (7.14%)

**Table 6:** Follow-up outcomes.

Outcome	Cases (%)
Disease-free, no recurrence	19 (67.89%)
Relapse	3 (10.71%)
Deaths	2 (7.14%)
No available data	4–2 MM, 2 refused treatment (14.28%)

**Table 7:** Summary of our institutional case series.

Parameter	Case 1	Case 2	Case 3
Age/Sex	43 M	79 F	47 M
Symptoms	Bilateral nasal obstruction, epistaxis (3 months)	Left nasal obstruction, epistaxis (2 years)	Right nasal obstruction (1 + year)
Clinical and Endoscopic Findings	Infiltrating, vegetative mass in the left nasal fossa and nasopharynx	Sessile, reddish mass, in the left nasal fossa	Sessile mass in the right nasal cavity
Imaging	No bone damage	No bone invasion	No bone lesion
Surgery	En bloc resection, endoscopic	En bloc resection, endoscopic	En bloc resection, endoscopic
Histopathology	Plasmacytoid cells, hemorrhage	Plasmacytoid cells, necrosis	Compact eosinophilic cells
Immunohistochemistry	CD38+, CD138+	CD79a+, CD138+, CD56+	CD138+
Radiation	-	40 Gy/3 weeks	44 Gy/1 month
Follow-up	1 year, no recurrence	2 years, no recurrence	18 months, no recurrence
Multiple Myeloma	Negative	Negative	Negative

## Data Availability

The raw data supporting the conclusions of this article will be made available by the authors on request.

## Abbreviations

The following abbreviations are used in this manuscript:

Full Term	Abbreviation
Extramedullary plasmacytoma	EMP
Multiple myeloma	MM
Solitary plasmacytoma of bone	SPB
Radiotherapy	RT
Chemotherapy	CHT
Magnetic resonance imaging	MRI
Computed tomography	CT
Cluster of differentiation	CD
Kappa light chains	KLCs
Lambda light chains	LLCs
Endoscopic nasal surgery	ENS
Complete blood count	CBC
Estimated glomerular filtration rate	eGFR
Sinonasal extramedullary plasmacytoma	SN-EMP
